# A Comprehensive Review of Tubulointerstitial Nephritis and Uveitis (TINU) Syndrome

**DOI:** 10.3390/biomedicines13020300

**Published:** 2025-01-26

**Authors:** Alexandra Bograd, Arnd Heiligenhaus, Stefan Reuter, Christoph Tappeiner

**Affiliations:** 1 Department of Ophthalmology, Pallas Kliniken, 4600 Olten, Switzerland; 2 Department of Ophthalmology at St. Franziskus Hospital, 48145 Münster, Germany; 3 Division of General Internal Medicine, Nephrology and Rheumatology, Department of Medicine D, University Hospital of Münster, 48149 Münster, Germany; 4 Department of Ophthalmology, University Hospital Essen, University of Duisburg-Essen, 45147 Essen, Germany; 5 Faculty of Medicine, University of Bern, 3008 Bern, Switzerland

**Keywords:** TINU syndrome, tubulointerstitial nephritis, uveitis, autoimmune diseases, corticosteroids, immunomodulatory therapies, biologic agents, pathophysiology, diagnosis, treatment

## Abstract

**Background:** Tubulointerstitial nephritis and uveitis (TINU) syndrome is a rare autoimmune disorder, characterized by acute tubulointerstitial nephritis and uveitis. It poses diagnostic challenges due to the mostly asynchronous onset of renal and ocular manifestations, as well as the variety of differential diagnoses. This review provides an overview of the epidemiology, pathogenesis, clinical features, diagnostic criteria, and management strategies. **Methods:** A comprehensive review of the peer-reviewed literature, including studies and case reports, was conducted. **Results:** The etiology of TINU syndrome involves an autoimmune reaction to renal and ocular antigens, leading to interstitial inflammation and tubular damage in the kidneys, and anterior uveitis with acute onset of flares. Diagnostic criteria based on ocular examination, laboratory parameters, and renal biopsy emphasize the need to exclude other systemic diseases. TINU syndrome accounts for approximately 2% of all uveitis cases. Primary treatment consists of corticosteroids, while immunomodulatory therapies (methotrexate, azathioprine, mycophenolate mofetil, or biologic agents) are reserved for refractory cases. Recurrence of uveitis appears to be more common than that of nephritis. **Conclusions:** TINU syndrome is rare and requires clinical suspicion for accurate diagnosis. Early diagnosis and initiation of treatment are crucial for achieving favorable outcomes. Advances in the understanding of its pathogenesis and treatment have improved patient outcomes. Further research is needed to investigate the underlying triggers and mechanisms in order to develop targeted therapies.

## 1. Introduction

Tubulointerstitial nephritis and uveitis (TINU) is a rare manifestation of predominantly bilateral non-granulomatous anterior uveitis associated with autoimmune acute interstitial nephritis (AIN). This clinical entity was first described in 1975 by Robert S. Dobrin as a combination of anterior uveitis, eosinophilic interstitial nephritis, and reversible renal failure [1]. To date, approximately 600 cases have been reported worldwide [2]. TINU syndrome, while a diagnosis of exclusion in the absence of other underlying diseases, is a distinct clinical entity characterized by a range of clinical findings. Given its potential for underdiagnosis, it is essential to understand its diagnostic criteria, varied clinical manifestations, and therapeutic strategies, which are the focus of this article. We herein discuss also the standard treatment approach with corticosteroids, and the use of conventional synthetic disease-modifying antirheumatic drugs (csDMARDs) in cases of persistent or recurrent disease. Additionally, we highlight the existing literature on the application of tumor necrosis factor (TNF)-alpha inhibitors. Our objective is to increase awareness of this rare but significant condition, provide insight into its pathophysiology, and propose a therapeutic algorithm based on current evidence, despite the absence of randomized controlled trials.

A comprehensive literature search was conducted using PubMed, including both established and recent publications from 1975 to 2024, with a particular emphasis on the most recent five-year period (2019–2024). The following search terms were applied: “tubulointerstitial nephritis and uveitis” or “TINU” or “acute interstitial nephritis” or “AIN” and “uveitis”. In a second step, the search was refined using additional terms such as “diagnostic criteria”, “epidemiology”, “pathophysiology”, “clinical signs” or “findings”, “therapy” or “treatment”, “differential diagnosis”, “beta-2 microglobulin”, “recurrence rate”, or “complications”.

## 2. Epidemiology and Risk Factors

TINU syndrome as an underlying cause of anterior uveitis is rare and probably often unrecognized, accounting for an estimated 1–2% of all acute bilateral uveitis cases [3]. The mean age of onset is 15 years (range 9–74 years) [3]. For a long time, it was believed that female patients were more frequently affected, with a ratio of 3:1 being stated [4]. However, more recent publications indicate a higher rate of male patients. [5,6]. Mackensen et al. reported that 60% of their TINU patients were male, highlighting the predominance of male over female involvement [5,6]. Currently, knowledge regarding predisposing factors remains limited [7]. In certain cases, previous infections or the use of specific medications, including antibiotics and non-steroidal anti-inflammatory drugs (NSAIDs), demonstrate a correlation with the condition. TINU syndrome has also been observed in patients with autoimmune diseases [8,9,10]. This is consistent with evidence that certain HLA class II haplotypes, such as HLA DR B1*0102, HLA DR A1*01, and HLA DR B1*05, may increase genetic susceptibility to TINU syndrome [4,11].

## 3. Etiology

TINU syndrome is an autoimmune reaction that targets the renal interstitium [3]. The exact cause remains unknown. It is thought to result from an immune-mediated response involving both cellular and humoral immunity. Environmental triggers, such as infections or medications, may activate an immune response in genetically predisposed individuals, then leading to inflammation in both the kidneys and eyes. Possible triggers include drug toxicity and hypersensitivity, especially in response to antibiotics (e.g., beta-lactams, glycopeptides, tetracyclines, tuberculostatics, sulfonamides, and fluoroquinolones) and NSAIDs [7,12,13]. Infections may also lead to immune dysregulation, triggering abnormal T-cell responses that can result in TINU syndrome [7]. The research literature discussed the cytotoxic response of antibiotics, as well as a humoral immune response involving antibody formation and binding to the tubular membrane. [14]. NSAIDs not only cause abrupt changes in kidney function, such as reducing blood flow, they can also trigger AIN [15]. Additionally, cyclooxygenase inhibition and the conversion of arachidonic acid to leukotrienes activate T-helper cells and release cytokines, which increases glomerular permeability [13,14].

## 4. Pathophysiology

Specific HLA alleles, notably HLA-DRB1*0102, HLA-DQA1*01, and HLA-DQB1*05, are linked to TINU in diverse populations [4,10,16]. These are HLA class II molecules that are responsible for presenting antigens on CD4-positive T-lymphocytes and regulating the activation of the adaptive immune response via B-cells [16]. The predisposition of certain HLA types suggests that individuals possessing these alleles are more susceptible to an overactive immune response to specific environmental triggers (probably differing in the various regions) such as infections or drug-related toxins [16]. Moreover, the present findings indicate significant genetic variations in the frequency of IL-10 alleles in patients with TINU syndrome, suggesting a disturbance in cytokine regulation [16].

**Figure 1:**
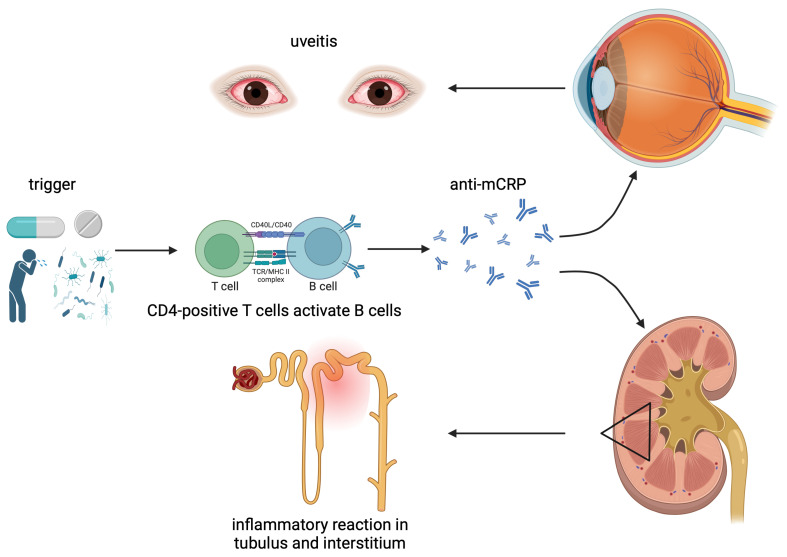
Pathophysiology of TINU syndrome. Drug-related toxicity or infections may trigger an immunological cascade, potentially activating B-cells and leading to the production of antibodies against modified C-reactive protein (anti-mCRP). These antibodies target mCRP proteins in the uvea, renal interstitium, or tubules, resulting in uveitis and acute interstitial nephritis. (Created in BioRender. Bograd, A. (2025) https://BioRender.com/z95m909, accessed on 22 January 2025).

Stimulated B lymphocytes produce immunoglobulin G antibodies against the modified C-reactive protein (anti-mCRP IgG) (Figure 1) [3,11,17]. The protein mCRP, as a target of this immune reaction, is a monomer of CRP and is produced under specific conditions such as altered pH, high urea, or low calcium concentrations [11]. Given that mCRP is present in normal tubular epithelial cells, interstitial cells, and endothelial cells of the uvea, the simultaneous antigen–antibody reaction in the renal interstitium, renal tubules, and uvea is explicable. [11]. Clinical observations confirm elevated serum anti-mCRP levels in patients diagnosed with TINU syndrome, as well as in patients with Sjögren’s syndrome and lupus nephritis [11,18].

Beta-2-microglobilin (β2M), a small molecular weight protein found on membranes in renal tubular cells, is involved in glomerular filtration, tubular reabsorption, degradation in tubular cells, and urinary secretion [19,20]. In patients with impaired tubular function, β2M serum levels may rise, correlating with the degree of renal insufficiency. Increased urinary levels of β2M indicate tubular dysfunction in tubulointerstitial nephritis and may serve as a biomarker for detecting renal involvement in TINU patients [20]. While elevated serum creatinine remains a common indicator of renal impairment, urinary β2M correlates with the histological grade of tubulointerstitial nephritis [21]. Increased urinary β2M levels reflect the extent of tubular dysfunction and disease severity [22]. Inflammatory processes result in cell destruction within the renal interstitium, leading to decreased tubular reabsorption and a reduced glomerular filtration rate. Consequently, urinary β2M levels may be elevated and serum creatinine levels may increase [20]. Additional laboratory findings may include leukocyturia, glycosuria, eosinophilia, anemia, mildly elevated liver enzymes, and an increased erythrocyte sedimentation rate and CRP levels. However, no laboratory findings are highly specific or pathognomonic for TINU syndrome. Kidney biopsy typically reveals tubulointerstitial edema and inflammatory cell infiltration, primarily composed of mononuclear cells, such as lymphocytes, plasma cells, and histiocytes. While eosinophils and noncaseating granulomas are commonly present, neutrophils may occasionally be observed.

## 5. Clinical Presentation

Nephritis may remain undetected for a long time, as renal symptoms frequently manifest as non-specific, which often results in them being inadvertently overlooked or misinterpreted [23]. In 98% of TINU patients, the initial suspicion of syndrome is raised by an ophthalmologist [4]. Prodromal B-symptoms include fever, weight loss, night sweats, fatigue, arthralgia, and myalgia [4]. The clinical findings of uveitis and AIN are presented in Table 1.

### 5.1. Ocular Symptoms and Presentation

In approximately 80% of cases, ocular inflammation is primarily confined to the anterior segment of the eye, such as the ciliary body, iris, and anterior chamber, with bilateral non-granulomatous anterior uveitis [31]. Granulomatous anterior involvement is less common (20% of TINU patients) [4,24,31]. Uveitis commonly flares with sudden onset, and symptoms of anterior segment inflammation may include ocular pain, eye redness, and photophobia [4]. Nonetheless, the results of the prospective study carried out by Saarela et al. demonstrated that in over 50% of cases, patients exhibited asymptomatic uveitis [32]. This suggests the possibility of a delay before the initial ophthalmological examination can be conducted.

### 5.2. Renal Symptoms

Patients can experience abdominal pain, skin rashes, and dys- and anuria [28]. Renal involvement is usually acute (acute kidney injury) and tends to improve within a few months with appropriate corticosteroid treatment. The renal function typically normalizes within 6–12 months [3,4].

### 5.3. Ocular Complications

Ocular complications, which can lead to significant visual morbidity and reduced quality of life, are observed in approximately 20% of TINU patients. Posterior synechiae is the most frequently reported complication, though cataract formation, glaucoma, optic disc swelling, cystoid macular edema, and chorioretinal scarring may also develop [4,5,7,33]. Secondary complications such as cataract, macular edema, and papillitis may occur in patients with uncontrolled uveitis [34]. However, intermediate and posterior uveitis have been reported in 5–10% of TINU patients, as well as rare cases of secondary choroidal neovascularization (CNV) [3,4,35]. Studies show a chronic course of uveitis with persistent inflammation or frequent relapses at intervals of less than three months in up to 50% of patients. [3,4].

### 5.4. Renal Complications

AIN may lead to significant kidney damage, if not treated; thus, in 40–60% of cases, AIN progressed to fibrosis, ultimately causing irreversible loss of kidney function [36].

## 6. Diagnosis

TINU syndrome typically presents with non-specific systemic symptoms such as fever, weight loss, malaise, and fatigue. These symptoms can easily be mistaken for other illnesses, disguising early diagnosis. Renal symptoms, such as flank pain, hematuria, and proteinuria, may occur but are often subtle. Eye involvement may present either concurrently with renal symptoms or months apart, or even before, leading to further diagnostic delays.

### 6.1. Diagnostic Criteria

**Figure 2:**
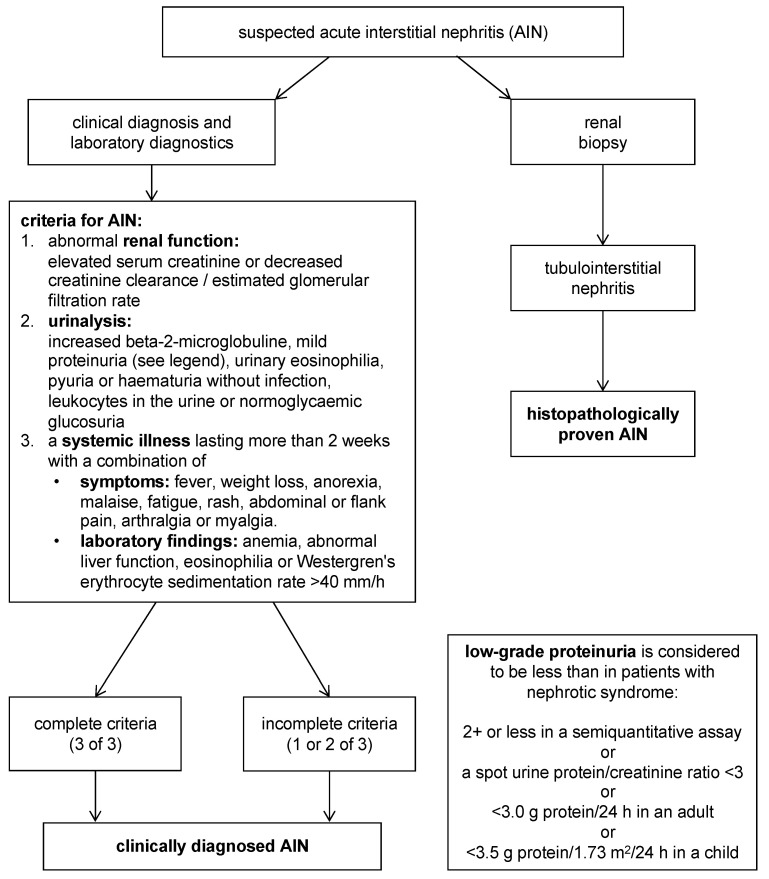
Diagnostic criteria for tubulointerstitial nephritis and uveitis syndrome, based on the publications of Mandeville et al. [4] and Bograd et al. [37].

Diagnostic criteria introduced by Mandeville et al. in 2001 (Figure 2) require the presence of both acute interstitial nephritis (AIN) and uveitis, in the absence of other systemic diseases known to be associated with AIN or uveitis [4]. Histopathological confirmation of AIN via renal biopsy is ideal but not always necessary if clinical criteria are fulfilled (Table 2). However, definite diagnosis may be obtained by renal biopsy.

The Standardization of Uveitis Nomenclature (SUN) Working Group identified classification criteria for TINU syndrome using machine learning applied to a database of 1083 anterior uveitis cases, including 94 TINU cases [38]. Key factors identified included anterior chamber inflammation combined with evidence of tubulointerstitial nephritis, defined by either a positive renal biopsy or nephritis markers such as elevated serum creatinine, abnormal urine analysis, and elevated urine β2M [38]. The method achieved high accuracy, with a misclassification rate of 1.2% in the training set and 0% in the validation set, demonstrating its potential utility in clinical and translational research.

### 6.2. Laboratory Parameters

Serum creatinine and β2M levels are sensitive screening parameters for TINU syndrome in young uveitis patients. The combined use of these tests enhances diagnostic accuracy by increasing the positive predictive value [38,39,40]. However, β2M lacks specificity, as its serum levels are also elevated in patients with chronic kidney disease and increase with declining glomerular filtration rate [41]. As a result, clinicians rarely include β2M assessment in routine diagnostic procedures for AIN.

Urine analysis may show non-specific findings, such as proteinuria, hematuria, glycosuria, and aminoaciduria, which indicate tubular dysfunction. Specific biomarkers of tubular damage, such as N-acetylglucosaminidase, may also be elevated in TINU patients [22,39].

### 6.3. Biopsy

Finally, a renal biopsy is the definitive diagnostic tool, revealing inflammatory cell infiltration (mainly lymphocytes and monocytes), tubular damage, and interstitial edema, with unaffected glomerular structures, and helping distinguish TINU from other glomerular diseases [22]. Newer techniques, such as diffusion-weighted magnetic resonance imaging (DW-MRI), may be useful for the early detection of TINU, offering a non-invasive alternative to biopsy in some patients [22,42].

A systematic approach to diagnose the renal component of TINU syndrome is recommended. Clinical assessment should first raise suspicion of AIN, particularly in patients presenting with systemic symptoms, such as flank pain or hematuria [43]. Urinalysis may reveal typical findings such as sterile pyuria, hematuria, and proteinuria. Blood tests are essential to evaluate inflammatory markers, eosinophilia, and elevated creatinine levels. Imaging studies, specifically renal ultrasound, can be useful to detect any kidney swelling or structural abnormalities [43,44]. In cases requiring further confirmation, a renal biopsy may be conducted, often revealing classic signs of AIN, including tubulointerstitial edema and mononuclear cell infiltration [45].

**Figure 3:**
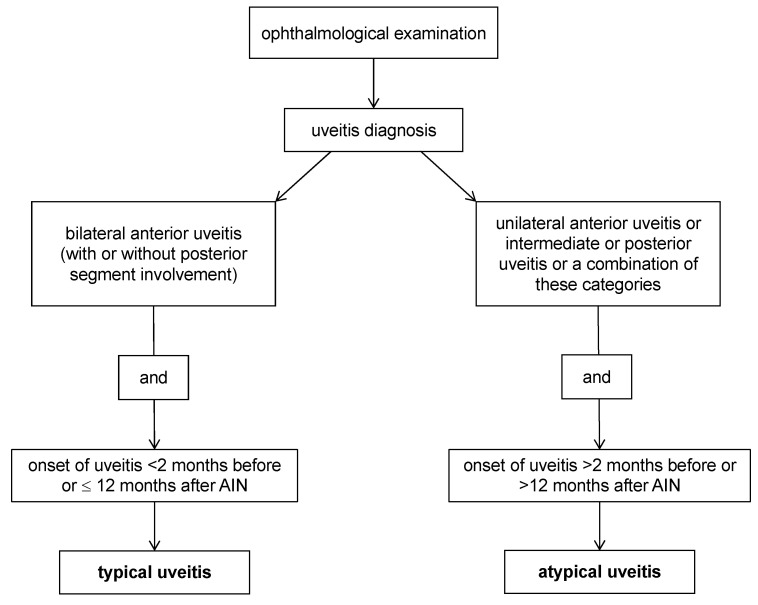
Definition of typical and atypical uveitis in TINU syndrome based on clinical presentation and timepoint of onset in relation to acute interstitial nephritis (AIN), according to Mandeville et al. [4] and Bograd et al. [37].

Uveitis in TINU patients is defined as typical or atypical based on clinical presentation and timepoint of onset in relation to AIN (Figure 3) [4,37].

The diagnosis of TINU syndrome should be considered in patients presenting with AIN and uveitis, regardless of whether the ocular involvement occurs concurrently with, or precedes or follows, the onset of AIN. Diagnostic confirmation is further strengthened by ruling out other identifiable causes and by characteristic renal biopsy findings, including mononuclear cell infiltration and, in some cases, granulomas. Although the presence of autoantibodies and elevated inflammatory markers, such as mCRP and ANA, may provide additional supporting evidence, they are not diagnostic on their own.

### 6.4. Differential Diagnoses

A variety of differential diagnoses should be considered, including diseases that can cause altered renal parameters in association with uveitis [4].

#### 6.4.1. Systemic Lupus Erythematosus (SLE)

SLE can manifest as tubulointerstitial nephritis and uveitis as part of its systemic manifestations, often involving other organs, such as the skin, joints, and serosa. The characteristic findings are positive antinuclear antibodies (ANAs), anti-dsDNA, and low complement levels, which allow differentiation from TINU [46].

#### 6.4.2. Behçet’s Disease (BD)

BD presents as recurrent uveitis and possible kidney involvement, although nephritis is less common. Other characteristics include oral and genital ulcers. Mucocutaneous symptoms, a positive pathergy test, and a positive HLA-B51 haplotype, can assist in the diagnostic process [47].

#### 6.4.3. Sjögren’s Syndrome

Sjögren’s syndrome is a systemic autoimmune disease primarily affecting the exocrine glands, leading to dry eyes and mouth, but it can also affect the renal system. Key findings include positive anti-Ro/SSA and anti-La/SSB antibodies, along with keratoconjunctivitis sicca and xerostomia [48].

#### 6.4.4. Infections

Certain infections, such as tuberculosis and syphilis, may present with uveitis and interstitial nephritis, resembling TINU syndrome. However, positive tests for tuberculosis (e.g., purified protein derivative test or interferon-gamma release assays) or syphilis (e.g., fluorescent treponemal antibody absorption test) will indicate an infectious etiology [49]. Epstein–Barr virus (EBV) is a common viral infection that can cause tubulointerstitial nephritis and ocular manifestations such as uveitis. Distinguishing features include mononucleosis, positive serology for EBV (elevated EBV IgM or IgG), and clinical features such as pharyngitis, lymphadenopathy, and elevated liver enzymes [50]. Infection must be ruled out before starting systemic corticosteroid or immunomodulatory treatment.

#### 6.4.5. Sarcoidosis

Sarcoidosis is a granulomatous multisystem disorder commonly affecting the eyes, lungs, and lymph nodes. The kidneys are affected in about one-third of patients, while ocular involvement (mostly granulomatous) occurs in approximately 25–60% of cases [51,52]. Non-caseating granulomas, pulmonary involvement (mediastinal lymphadenopathy), and elevated serum angiotensin-converting enzyme (ACE) levels can help distinguish sarcoidosis from TINU [31].

#### 6.4.6. ANCA-Associated Vasculitis (AAV)

AAV can manifest with renal involvement (glomerulonephritis or interstitial nephritis) and ocular manifestations, such as uveitis [53]. Both TINU and AAV can present with renal and ocular involvement; however, AAV is characterized by systemic vasculitis affecting small vessels, such as capillaries, venules, and arterioles, leading primarily to renal and respiratory manifestations [54,55]. The presence of anti-neutrophil cytoplasmic antibodies (ANCA), especially PR3-ANCA or MPO-ANCA, is a key factor [56].

#### 6.4.7. Granulomatous Polyangiitis (GPA, Formerly Wegener’s Disease)

GPA, a subtype of AAV, can affect both the kidneys (glomerulonephritis or tubulointerstitial nephritis) and eyes (scleritis or uveitis). It frequently involves the respiratory tract, causing nasal or sinus problems. PR3-ANCA positivity is common, and biopsies often reveal granulomatous inflammation. Nasal, pulmonary, and cutaneous involvement are characteristic indicators [57].

#### 6.4.8. Hyper-IgG4 Associated Diseases

This fibroinflammatory disease can affect multiple organs, including the kidneys and the eyes [58,59]. It may cause tubulointerstitial nephritis, idiopathic orbital inflammation (orbital pseudotumor), and anterior uveitis, mimicking TINU syndrome [60]. Elevated serum IgG4 levels and histological evidence of IgG4-positive plasma cell infiltration help to differentiate this condition [61].

#### 6.4.9. Juvenile Idiopathic Arthritis (JIA)-Associated Uveitis

This manifestation of recurrent or chronic anterior uveitis typically occurs in children and adolescents under the age of 16 [62]. It is frequently associated with oligoarticular arthritis [62]. The absence of renal involvement in JIA-associated uveitis effectively excludes TINU syndrome as a differential diagnosis.

## 7. Treatment

Although renal inflammation in TINU syndrome is generally self-limiting, it requires early and appropriate treatment to ensure full recovery and prevent potential chronic complications [63].

### 7.1. Systemic Corticosteroids

For the treatment of uveitis, it is recommended that both topical and systemic corticosteroids (1 mg/kg/day for 3–6 months, depending upon the response) are initiated promptly [64]. Tapering is usually based on the response to therapy and may be gradually reduced on a weekly basis under monitoring eye inflammation. This therapeutic strategy typically achieves uveitis inactivity with a significantly reduced relapse rate [65]. Approximately 70% of patients exhibit a favorable response to corticosteroid monotherapy for renal involvement [63].

### 7.2. Immunomodulatory Therapy

In cases where the renal manifestations become chronic or do not respond adequately to corticosteroids alone, additional systemic therapy with disease-modifying anti-rheumatic drugs (DMARDs, e.g., azathioprine) may be necessary [63]. Systemic corticosteroid-sparing immunomodulatory therapy should be considered in the setting of frequent flares and chronicity, after ruling out possible contraindications and in strict interdisciplinary consultation with the treating nephrologist. A review of the current literature suggests that methotrexate (MTX), mycophenolate mofetil (MMF), and azathioprine are regarded as effective and safe for managing TINU syndrome [22,66,67,68]. However, the evidence is derived solely from case series and uncontrolled studies, as no randomized controlled trials assessing their efficacy are currently available.

Recent findings indicate that TNF-alpha inhibitors may serve as a viable option for cases of persistent or recurrent inflammation not responding adequately to systemic corticosteroids and csDMARDs [69]. TNF-alpha inhibitors, such as adalimumab and infliximab, are primarily used in cases of uveitis unresponsive to monotherapy with csDMARDs, including MTX or MMF [25,33,70]. These agents have demonstrated efficacy in controlling inflammation and reducing recurrence rates, making them a valuable therapeutic option for patients with refractory disease and as a steroid-sparing strategy [70].

**Figure 4:**
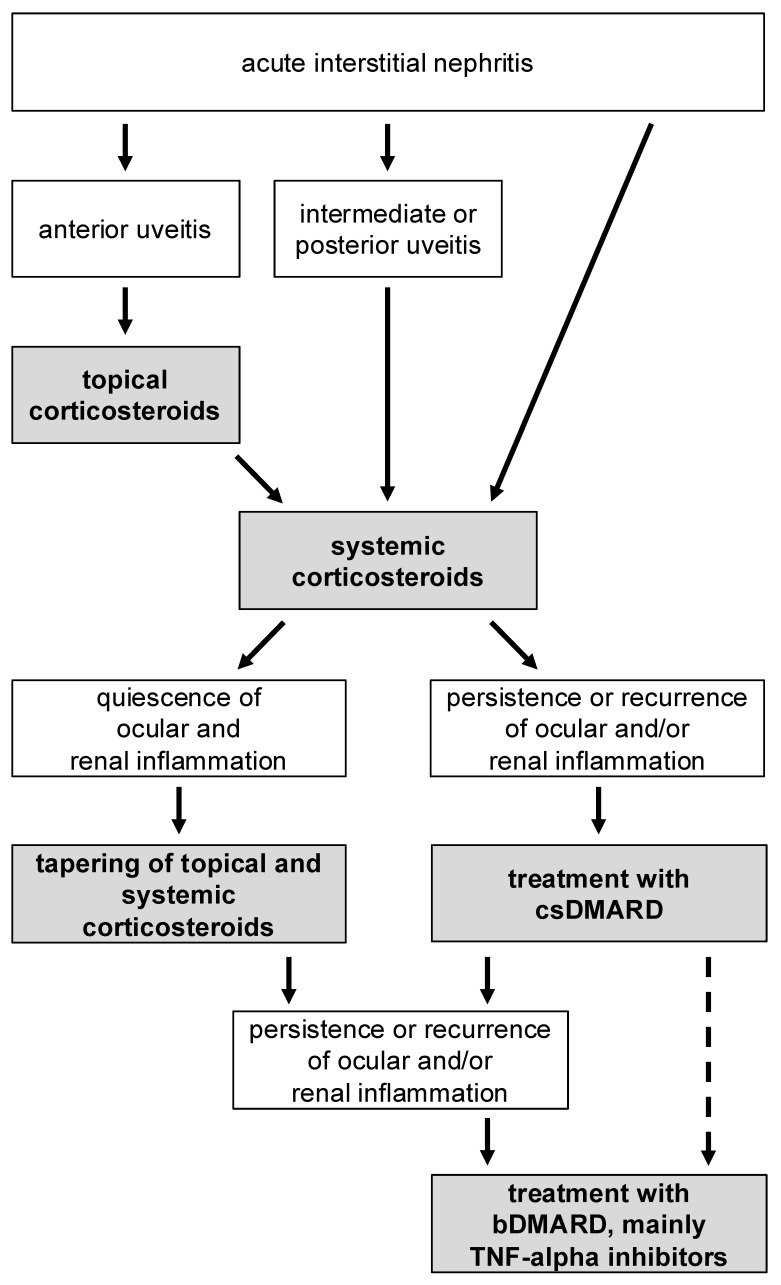
Treatment algorithm for TINU syndrome. Initial management of anterior uveitis involves the use of topical corticosteroids (e.g., initially administered hourly, followed six times daily, with a gradual reduction by one drop per week). Following confirmation of TINU syndrome and exclusion of underlying infectious diseases, systemic corticosteroids (e.g., 1 mg/kg body weight/day) should be initiated. If both renal and ocular inflammation are effectively controlled, the therapy can be tapered gradually. In cases of recurrence or insufficient response to systemic corticosteroids, escalation to conventional synthetic disease-modifying antirheumatic drugs (csDMARDs) is recommended. If necessary, biologic DMARDs (bDMARDs), particularly TNF-alpha inhibitors, may be considered.

In the absence of randomized prospective trials, there are no uniform recommendations for the choice of agent; therefore, general principles for corticosteroid-sparing and immunomodulatory treatment of chronic inflammatory diseases should be followed [16,70]. Nowadays, biologic agents are used more commonly to achieve long-term disease remission [33]. The proposed algorithm, illustrated in Figure 4, outlines a therapeutic approach derived from the prevailing literature and current treatment recommendations for anterior uveitis associated with TINU syndrome. This figure aims to provide clinicians with a clear and practical framework for managing this condition effectively.

### 7.3. Long-Term Prognosis

With prompt initiation of treatment, TINU syndrome generally has a favorable prognosis. Nonetheless, the potential for long-term renal and ocular complications should be considered. According to Jahnukainen et al., permanent renal dysfunction persists in approximately 15% of patients, and chronic uveitis remains in up to 33% of cases, and related eye complications in 21% of cases [4,71].

## 8. Conclusions

TINU syndrome is a rare and often overlooked autoimmune disease. Because it is uncommon and presents with variable symptoms and clinical signs, combined with the potentially time-shifted onset of ocular and kidney disease, it is likely to be underdiagnosed. Early detection and treatment are critical for the prevention of long-term complications. Thorough screening procedures are crucial in identifying TINU syndrome, particularly in young patients with otherwise unclassified uveitis. A comprehensive diagnostic approach, including exclusion of possible differential diagnoses, ensures appropriate treatment, which typically includes systemic and topical corticosteroids and interdisciplinary collaboration between ophthalmologists and nephrologists. In chronically active or severe cases, DMARDs might be required. While renal symptoms generally improve with corticosteroid therapy, managing ocular involvement may be more challenging to prevent secondary complications and visual impairment. Further studies are needed to standardize treatment protocols and improve patient outcomes in this complex multi-organ disease. Specifically, there is a need for systematic diagnostic approaches to identify TINU in cases of uveitis, multicenter studies focusing on therapeutic strategies and the evaluation of prognostic biomarkers, and prospective studies on the efficacy and impact of biologic therapies.

## Figures and Tables

**Table 1:** Clinical findings of uveitis and acute interstitial nephritis in TINU syndrome.

**Uveitis [3,4,5,16,24,25,26,27]**	**Acute Interstitial Nephritis [26,27,28,29,30]**
ciliary injection	skin rash
keratic precipitates (fine or mutton fat)	renal failure and nephrotic syndrome
anterior chamber cells	high levels of serum creatinine
posterior synechiae	dysuria and anuria
iris nodules	eosinophilia
vitreous cells	elevated β2-microglobuline
chorioretinal lesions	proteinuria
optic disc swelling	hematuria
macular edema	leukocyturia
retinal vasculitis	interstitial fibrosis
posterior scleritis	elevated inflammatory parameters
choroidal neovascularization	tubulointerstitial inflammation

**Table 2:** Diagnostic criteria defining probable, possible, or definite TINU syndrome, based on Mandeville et al. [4] and Bograd et al. [37].

TINU Syndrome	AIN	Uveitis
**definite**		
either	AIN diagnosed by histopathology	typical uveitis
or	clinically diagnosed AIN (complete criteria)	typical uveitis
**probable**		
either	AIN diagnosed by histopathology	atypical uveitis
or	clinically diagnosed AIN (incomplete criteria)	typical uveitis
**possible**		
	clinically diagnosed AIN (incomplete criteria)	atypical uveitis
